# The effect of targeted hyperoxemia in a randomized controlled trial employing a long-term resuscitated, model of combined acute subdural hematoma and hemorrhagic shock in swine with coronary artery disease: An exploratory, hypothesis-generating study

**DOI:** 10.3389/fmed.2022.971882

**Published:** 2022-08-22

**Authors:** Thomas Datzmann, David Alexander Christian Messerer, Franziska Münz, Andrea Hoffmann, Michael Gröger, René Mathieu, Simon Mayer, Holger Gässler, Fabian Zink, Oscar McCook, Tamara Merz, Angelika Scheuerle, Eva-Maria Wolfschmitt, Timo Thebrath, Stefan Zuech, Enrico Calzia, Pierre Asfar, Peter Radermacher, Thomas Kapapa

**Affiliations:** ^1^Institut für Anästhesiologische Pathophysiologie und Verfahrensentwicklung, Universitätsklinikum Ulm, Ulm, Germany; ^2^Klinik für Anästhesiologie und Intensivmedizin, Universitätsklinikum Ulm, Ulm, Germany; ^3^Transfusionsmedizinische und Hämostaseologische Abteilung, Universitätsklinikum Erlangen und Friedrich-Alexander-Universität (FAU) Erlangen-Nürnberg, Erlangen, Germany; ^4^Klinik fuür Neurochirurgie, Bundeswehrkrankenhaus Ulm, Ulm, Germany; ^5^Klinik fuür Anästhesiologie, Intensivmedizin, Notfallmedizin und Schmerztherapie, Bundeswehrkrankenhaus Ulm, Ulm, Germany; ^6^Sektion Neuropathologie, Institut für Pathologie, Universitätsklinikum Ulm, Ulm, Germany; ^7^Département de Médecine Intensive – Réanimation et Médecine Hyperbare, Centre Hospitalier Universitaire, Angers, France; ^8^Klinik für Neurochirurgie, Universitätsklinikum Ulm, Ulm, Germany

**Keywords:** traumatic brain injury, multimodal brain monitoring, GFAP, MAP-2, protein S100β, neuron-specific enolase, mitochondrial respiration, oxidative stress hyperoxemia after acute subdural hematoma and hemorrhagic shock

## Abstract

Controversial evidence is available regarding suitable targets for the arterial O_2_ tension (P_a_O_2_) after traumatic brain injury and/or hemorrhagic shock (HS). We previously demonstrated that hyperoxia during resuscitation from hemorrhagic shock attenuated cardiac injury and renal dysfunction in swine with coronary artery disease. Therefore, this study investigated the impact of targeted hyperoxemia in a long-term, resuscitated model of combined acute subdural hematoma (ASDH)-induced brain injury and HS. The prospective randomized, controlled, resuscitated animal investigation consisted of 15 adult pigs. Combined ASDH plus HS was induced by injection of 0.1 ml/kg autologous blood into the subdural space followed by controlled passive removal of blood. Two hours later, resuscitation was initiated comprising re-transfusion of shed blood, fluids, continuous i.v. noradrenaline, and either hyperoxemia (target P_a_O_2_ 200 – 250 mmHg) or normoxemia (target P_a_O_2_ 80 – 120 mmHg) during the first 24 h of the total of 54 h of intensive care. Systemic hemodynamics, intracranial and cerebral perfusion pressures, parameters of brain microdialysis and blood biomarkers of brain injury did not significantly differ between the two groups. According to the experimental protocol, P_a_O_2_ was significantly higher in the hyperoxemia group at the end of the intervention period, i.e., at 24 h of resuscitation, which coincided with a higher brain tissue PO_2_. The latter persisted until the end of observation period. While neurological function as assessed using the veterinary Modified Glasgow Coma Score progressively deteriorated in the control group, it remained unaffected in the hyperoxemia animals, however, without significant intergroup difference. Survival times did not significantly differ in the hyperoxemia and control groups either. Despite being associated with higher brain tissue PO_2_ levels, which were sustained beyond the intervention period, targeted hyperoxemia exerted neither significantly beneficial nor deleterious effects after combined ASDH and HS in swine with pre-existing coronary artery disease. The unavailability of a power calculation and, thus, the limited number of animals included, are the limitations of the study.

## Introduction

Post-traumatic mortality depends on the presence of hemorrhagic shock (HS) and/or traumatic brain injury (TBI). Hemorrhagic shock accounts for 30% of trauma-related mortality (1), TBI is the most prevalent cause of death after polytrauma (2).

During HS, supplemental O_2_ is used because increasing the amount of physically dissolved O_2_ during blood loss-related impairment of O_2_ transport may faster repay a tissue O_2_ debt (3, 4). Despite its vasoconstrictor properties (3, 4), pure O_2_ ventilation improved tissue PO_2_ during HS (5). After near-lethal hemorrhagic shock, pure O_2_ ventilation also attenuated organ dysfunction (6). However, hyperox(em)ia enhances reactive oxygen species (ROS) formation, especially during I/R and/or hypoxia/re-oxygenation, e.g., resuscitation from trauma-and-hemorrhage (3, 4). After TBI, any hyperoxemia-related vasoconstriction may exert beneficial effects due to reduced intracranial pressure without compromised tissue oxygenation.

Current recommendations suggest that P_a_O_2_ > 300 mmHg (40 kPa) should be avoided (3, 4), but a putative “optimal level” after TBI and/or HS remains open: in trauma patients, retrospective analyzes showed that P_a_O_2_ > 150 and > 200 mmHg, respectively, within the first 24 h coincided with improved survival (7, 8). Moreover, P_a_O_2_≈150–250 mmHg during the first 24 h of intensive care unit (ICU) stay were associated with the lowest mortality and the best neurological outcome after severe TBI (9).

Therefore, this study was to investigate the impact of targeted hyperoxemia in a randomized, controlled, resuscitated, large animal model of combined acute subdural hematoma (ASDH) -induced TBI and HS over 56 h post-injury. The main outcome parameters were whether this approach (i) ameliorates brain tissue oxygenation, (ii) improves neurologic function, and (iii) is safe with respect to parameters of oxidative stress.

## Materials and methods

### Animals and preparations

After ethical approval by the Animal Care Committee of the Universität Ulm and the Federal Authorities for Animal Research (Regierungspräsidium Tübingen, #1316) experiments were conducted in adherence to the European Union Directive 2010/63/EU on the protection of animals used for scientific purposes. 15 adult Familial hypercholesterolemia-Bretoncelles-Meishan (FBM (10) pigs (median[interquartile range] body weight 63[56; 71]kg; age 38[36; 41] months) of either sex (8 castrated males, 7 females) were used. This pig strain is a cross-bread of Rapacz farm pigs homozygous for the R84C low density lipoprotein receptor mutation with the smaller Meishan and Bretoncelles strains, and at the age of at least 9 – 12 months, it presents with atherosclerosis and coronary artery disease due to a cholesterol-enriched diet administered for at least 3 – 4 months (11). The animals in our study had received the atherogenic diet for at least 9 months, and we had previously confirmed typical coronary artery lesions in animals of similar age that had undergone this treatment (12, 13). FBM swine were studied because ASDH as a major contributor to TBI (14) assumes particular importance in the elderly and co-morbid patient (15). Moreover, in previous experiments investigating therapeutic interventions during hemorrhage-and-resuscitation we had shown that either hyperoxemia or infusion of Na2S2O3 had exerted organ-protective effects in swine with coronary artery disease (6, 16), while no benefit was obtained in cardiovascular healthy animals undergoing the same treatment protocol (17, 18). Due to one drop-out because of uncontrollable bleeding during the surgical instrumentation data refer to 14 animals.

Anesthesia and (neuro)surgical instrumentation have been described in detail previously (10). Briefly, during the 12 h preceding the experiments, animals received a nutritional solution (Fresubin^®^, Fresenius Kabi) and had free access to water. This approach is based on our previous experiments (6, 10, 16, 18): administration of a nutritional solution instead of a regular diet allows for feeding the animals and thus avoiding stress to hunger until the induction of anesthesia while at the same time the potential danger of regurgitation of solid food particles and their aspiration into the trachea is prevented during induction of anesthesia and prior to endotracheal intubation. Prior to induction of anesthesia, premedication consisted of intramuscular 5 mg/kg azaperone and 1–2 mg/kg midazolam. Anesthesia was induced with intravenous i.v. propofol (1–2 mg/kg) and ketamine (1 mg/kg). Pigs were intubated endotracheally and mechanically ventilated (ventilator settings: tidal volume 8 ml/kg, respiratory rate 8–12 breaths/minute adapted to an arterial PCO_2_ [PaCO_2_] = 35–40 mmHg, inspiratory/expiratory ratio [I/E] = 1:1.5, fraction of inspiratory O_2_ [F_I_O_2_]) = 0.3, positive end-expiratory pressure [PEEP] = 10 cmH_2_O to prevent formation of atelectasis). Anesthesia was maintained with continuous i.v. propofol (10 mg/kg/h) and remifentanil (5 μg/kg initial bolus, followed by 15–20 μg kg^–1^ h^–1^). A balanced electrolyte solution (10 ml kg^–1^ h^–1^, Jonosteril 1/1^®^, Fresenius Kabi) was infused as maintenance fluid. After surgical exposure, a 9-F catheter sheath was inserted into the right femoral vein for placement of a 4-lumen venous catheter (Arrow International). Both femoral arteries were exposed for placement of a 4-F PiCCO catheter (PULSION Medical Systems) for continuous cardiac output, pulse pressure and stroke volume variation measurement, and a 10-F catheter for blood sampling and passive blood removal to induce HS. A catheter was placed in the urinary bladder via midline mini-laparotomy. Thereafter, the animal was turned into the prone position, the skull was exposed, and a craniotomy was drilled over the left and right parietal cortices (10). After exposing the dura, a small incision was made, and a catheter was inserted approximately 5 mm into the subdural space. On the contralateral side, a similar burr hole was placed. Afterward, microdialysis catheters and multimodal brain monitoring probes (Neurovent-PTO^§^, Raumedic) were inserted about 10–15 mm into the brain parenchyma in both hemispheres. The multimodal probes were placed under visual control for intracranial pressure (ICP), brain tissue O_2_ partial pressure (P_bt_O_2_), and temperature measurements. The probes were calibrated before insertion according to the manufacturer’s specifications. After placement, all catheters were allowed to equilibrate for about 1 h, and recording was started when P_bt_O_2_ values were stable. At the end of the neurosurgical procedure, the burr holes were closed using bone wax, which also served for catheter, microdialysis and probe fixation. Bilateral neurosurgery was performed to avoid sham experiments, i.e., to comply with the 3R principle the hemisphere without ASDH served as the control for the hemisphere with ASDH. During surgery, hydroxyethyl starch 6%–130/0.42 (Vitafusal^§^, Serumwerk, Bernburg) was used to maintain pulse pressure variation, with a maximum dose of 30 ml/kg according to the manufacturer’s specifications.

### Experimental protocol

**FIGURE 1:**
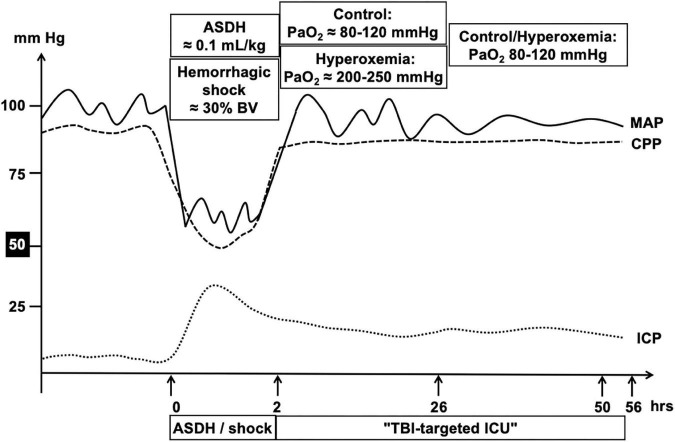
Schematic summary of the experimental design. After instrumentation and a resting period, ASDH was induced by injection of 0.1 ml/kg autologous blood into the subdural space. Immediately after induction of ASDH, hemorrhagic shock was initiated by passive removal of blood targeting 30% of the calculated blood volume. Blood removal was slowed down if necessary to maintain cerebral perfusion pressure (CPP), i.e., the difference between the simultaneously measured mean arterial (MAP) and intracranial (ICP) pressures, ≥ 50 mmHg. At 2 h of combined ASDH and hemorrhagic shock, resuscitation was initiated comprising re-transfusion of shed blood, fluid resuscitation, and continuous i.v. noradrenaline titrated to maintain MAP at pre-shock levels and CPP ≥ 75 mmHg over a maximum of 54 h. Animals were randomly assigned to control (normoxemia, P_a_O_2_ ≈ 80 – 120 mmHg throughout the whole observation period) vs. targeted hyperoxemia (P_a_O_2_ ≈ 200 – 250 mmHg during the first 24 h of treatment, thereafter similar to the control group).

Figure 1 shows the experimental protocol. In order to mimic the clinical situation where resuscitation measures are initiated only after trauma-and-hemorrhage, maintenance fluid infusion rate was reduced to 100 ml/h, ventilator settings were tidal volume 8 ml/kg, PEEP = 0 cmH_2_O, I/E ratio = 1:2, F_I_O_2_ = 0.21. Thereafter, 0.1 ml/kg body weight of autologous blood was injected over 15 min via the subdural catheter using a syringe pump. This subdural blood volume was injected because ≈10% of the intracranial volume represents the threshold for supra-tentorial volume tolerance (19). Immediately thereafter, HS was initiated by passive removal of blood over 30 min via the 10F-arterial catheter targeting 30% of the calculated blood volume (6, 16). The removed blood was stored at 4–8°C in citrate-anticoagulated bags (6, 16). Blood removal was slowed if necessary to maintain cerebral perfusion pressure (CPP), i.e., the difference between mean arterial pressure (MAP) and ICP, ≥ 50 mmHg. After 105 min of HS, i.e., 2 h of combined ASDH and HS, resuscitation was initiated comprising re-transfusion of shed blood within 30 min, fluid resuscitation (20 ml kg^–1^ h^–1^; reduced to 10 ml kg^–1^h^–1^ if venous pressure > 16 mmHg), and continuous i.v. noradrenaline titrated to maintain MAP at pre-shock levels and CPP ≥ 75 mmHg over a maximum of 54 h (20). Temperature management aimed to achieve brain normothermia, i.e., external cooling with ice-cold water-filled bags was applied if brain temperature reached 39°C. Animals were randomly assigned to control (normoxemia, P_a_O_2_ ≈ 80 – 120 mmHg throughout the whole observation period) vs. targeted hyperoxemia (P_a_O_2_ ≈ 200 – 250 mmHg during the first 24 h of treatment, thereafter similar to the control group).

### Measurements and calculations

Measurements of systemic hemodynamics and gas exchange, arterial blood sampling for P_a_O_2_, PaCO_2_, acid-base-status, electrolyte (N^+^, K^+^) levels and metabolic parameters (lactate, glucose, urea) were performed together with ICP, CPP, P_bt_O_2_, and brain temperature at baseline, i.e., before initiation of ASDH and HS, at the end of the 2-h of combined ASDH and HS, and at 24 and 48 h of treatment. At baseline as well as at 24 and 48 h of treatment, anesthesia depth was reduced by transitorily stopping the continuous i.v. propofol and remifentanil until adequate spontaneous breathing had resumed for 30 min, and neurological function was assessed using a swine-adapted, veterinary Modified Glasgow Coma Scale (MGCS) as described in detail previously (10). Thereafter, continuous i.v. anesthesia was resumed. Upon initiation of resuscitation, baseline ventilator settings were reinstalled, except for F_I_O_2_: during the first 24 h of treatment, after drawing ballots from a box, animals were randomly assigned to hyperoxemia (P_a_O_2_ = 200–250 mmHg) vs. control (normoxemia, P_a_O_2_ = 80–120 mmHg). This P_a_O_2_ target window was chosen, because it had coincided with the lowest mortality and the best neurological outcome after severe TBI (16). Thereafter, both groups were treated with the same ventilator settings until the end of the experiment. After 54 h of intensive care, i.e., after treatment had been continued for another 6 h after the last neurological assessment, the pigs were euthanized with KCl after further anesthesia deepening. Animals were euthanized before the end of the programmed 54-h period if (i) CPP < 60 mmHg despite maximum vasopressor dose, limited to a heart rate of ≤ 160/min to prevent tachycardia-induced myocardial injury, and/or the presence of (ii) refractory acute respiratory failure with P_a_O_2_ < 60 mmHg at F_I_O_2_ = 1.0, and/or (iii) anuric acute kidney failure with K^+^ > 6 mmol/L. Target P_a_O_2_ values during the hyperoxemia period never required F_I_O_2_ levels > 0.4, values during the control periods were always achieved using 0.23 < F_I_O_2_ < 0.3, and kaliemia always remained below 4 mmol/L (see Supplementary Table 1). Hence, all experiments terminated pre-schedule were due to sudden ICP increases and a refractory fall of CPP < 60 mmHg. To extrapolate the “missing values” for MGCS at the respective time points, in these animals the minimum MGCS score possible (= 3) was used.

Intracerebral tissue metabolites were determined using microdialysis (CMA 600 Microdialysis Analyzer^®^, CMA/Microdialysis AB) (10). Microdialysis catheters (70 microdialysis bolt catheter^®^, M Dialysis AB) were perfused (perfusion fluid, CMA/Microdialysis AB) by a microdialysis pump (CMA/102 microdialysis pump^®^, CMA/Microdialysis AB), and after calibration according to the manufacturer, microdialysate samples were collected in microvials (M Dialysis AB) over 3 h (i.e., from 1 h before until 2 h after each measurement time point) and immediately analyzed for glutamate, lactate, pyruvate, and glucose.

Serum concentrations of cytokines (interleukin-6 and 10) and the “brain injury markers” S100β (21), microtubule-associated protein 2 (MAP-2) (22), glial fibrillary acidic protein (GFAP) (22), and neuron-specific enolase (NSE) (21) were determined using commercially available porcine-specific kits as described previously (10, 16).

Immediately after sampling arterial whole blood samples were analyzed for superoxide anion (O_2_⋅^–^) concentrations by electron spin resonance (ESR) as described previously (23). Briefly, whole blood freshly drawn was mixed with the O_2_⋅^–^-specific spin probe 1-Hydroxy-3-methoxycarbonyl-2,2,5,5-tetramethylpyrrolidine and the metal chelators deferoxamine-methanesulfonate and diethyldithiocarbamic-acid in Krebs-Hepes-Buffer (all chemicals from Noxygen). After transferral to a glass capillary tube and incubation, the sample was analyzed using an ESR spectrometer (EMXnano^®^, Bruker) equipped with a temperature controller (BIO-III^®^, Noxygen). Averaging three scans, O_2_⋅^–^ concentrations were derived from comparison of the sample spectrum amplitude with that of standard dilution series of the stable radical 1-hydroxy-3-methoxycarbonyl-2,2,5,5-tetramethylpyrrolidine after subtraction of blank solution values.

At the end of the experiment, immediate *post mortem* brain tissue specimens from regions adjacent to the brain area next to the ASDH ipsilateral and corresponding contralateral area were analyzed for mitochondrial respiratory activity using “high-resolution respirometry” (Oxygraph-2K^®^, Oroboros Instruments) as described previously (10, 23). After supplementation of substrates for complexes I, II and ADP, the oxidative phosphorylation (coupled state, OXPHOS) was assessed. Maximal respiratory capacity of the electron transfer system (ETS) in the uncoupled state was measured after addition of 4-(trifluoromethoxy)phenylhydrazone. Data reported is normalized for tissue wet weight.

### Data analysis

We had predefined MGCS as the main outcome parameter. The MGCS had already been validated by others in dogs (Lit Platt) (24). Moreover, we have previously used the MGCS in swine undergoing ASDH alone without hemorrhagic shock and subsequent ICU care for 54 h (10). In that study, the absence of hemorrhagic shock allowed for maintenance of CPP and P_bt_O_2_ throughout the whole observational period, which prevented impairment of tissue energy metabolism and, subsequently, major neurological dysfunction. Consequently, a power analysis on the possible effect of hyperoxemia after ASDH *plus* hemorrhage was not feasible based on the available data, and, hence, we did not calculate a number of animals to include to observe a difference in GCS. Therefore, the Animal Care Committee of the Universität Ulm and the Federal Authorities for Animal Research (Regierungspräsidium Tübingen) deemed our study as “exploratory” allowing for *n* = 7 per group only. Consequently, we did not calculate a number of animals to include to observe a difference in GCS. Survival was analyzed using a Kaplan-Meyer-graph followed by Log-rank (Mantel-Cox) Test. Experimental data were considered to be non-parametric. Intergroup comparison was conducted with a Mann-Whitney *U*-test. Kruskal-Wallis test with *post hoc* Dunn’s multiple comparison was used for multiple within-group testing. Data in the figures is presented as individual data points as well as median and interquartile range. Table data is reported as median and interquartile range. Statistical analysis was conducted with GraphPad Prism5^®^ (GraphPad Software Inc., San Diego, CA, United States).

## Results

### Survival

**FIGURE 2:**
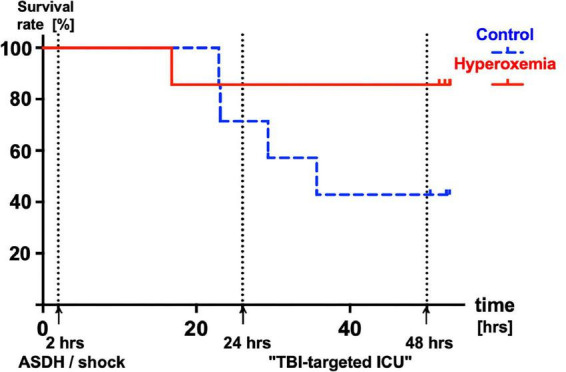
Kaplan-Meyer survival analysis of control (P_a_O_2_ ≈ 80 – 120 mmHg, *n* = 7, broken blue line) and animals treated with targeted hyperoxemia (P_a_O_2_ ≈ 200 – 250 mmHg, *n* = 7, solid red line) during the first 24 h of resuscitation. Median (interquartile range) survival times 54 (54; 54) and 36 (26; 54) hours in the hyperoxemia and control groups, respectively (*p* = 0.11).

Median survival times after initiation of resuscitation were 54 (54; 54) and 36 (26; 54) hours in the hyperoxemia and control groups and did not differ significantly between groups (*p* = 0.11) (Figure 2). No matter the group assignment, all experiments terminated pre-schedule were due to sudden ICP increases and consecutive fall of CPP < 60 mmHg.

### Hemodynamics, gas exchange, acid-base state and metabolism

Supplementary Table 1 summarizes the parameters of systemic hemodynamics, gas exchange, acid-base status, and metabolism. None of these parameters showed any significant intergroup difference throughout the experiment, except for significantly lower glycemia and hemoglobin content values in the hyperoxemia animals at the end of the 2 h of combined ASDH plus HS. Severity of shock was also comparable, the amount of blood withdrawn and the noradrenaline infusion rates needed to achieve hemodynamic targets significantly differed. Neither during the first 24 h (i.e., during the targeted hyperoxemia period) nor during the second half of the observation period diuresis showed any significant intergroup difference.

### Brain perfusion, oxygenation and microdialysis

**FIGURE 3:**
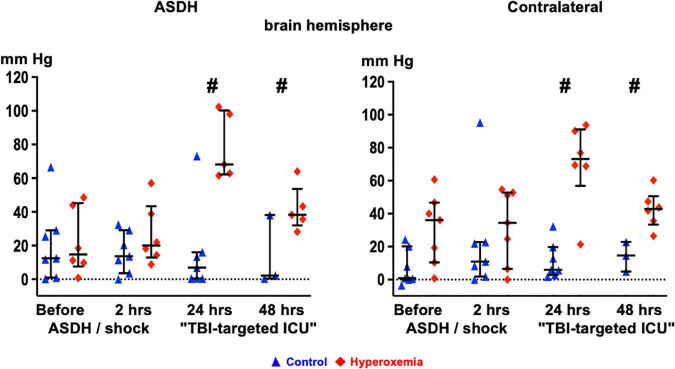
Time course of brain tissue O_2_ partial pressure (P_bt_O_2_) in the blood-injected (“ASDH”, left panel) and the contralateral (right panel) brain hemisphere in the control animals (blue triangles) and the targeted hyperoxemia group (red diamonds). Presented are individual data points, median and interquartile range; # denotes *p* < 0.05 vs. control. Note: At 24 h of “TBI-targeted ICU care” two control animals had already died. However, since these animals had to be euthanized at less than 2 h before the scheduled time point at 24 h of ICU care, the immediate pre-mortal P_bt_O_2_ values recorded at that time point were used as readings for “24 hours of TBI-targeted ICU care”.

Table 1 and Figure 3 show the parameters of brain perfusion, oxygenation and microdialysis. After combined ASDH and HS, ICP progressively rose and CPP fell until the end of the experiment. Figure 3 shows that target hyperoxemia was associated with significantly higher P_bt_O_2_ levels at the end of the 24-h intervention period both in the blood-injected and in the contralateral hemisphere, and this effect persisted until the end of the experiment. While microdialysis measurements did not show any significant effect for lactate or pyruvate, glucose concentrations progressively dropped, however, without intergroup difference. Glutamate concentrations also progressively declined in the hyperoxemia-group, while they remained unchanged in the control group (Table 1). Ultimately, in the hyperoxemia-group, this coincided with well-maintained neurological function, as suggested by the unaffected MGCS. In contrast, MGCS progressively fell in the control animals until the end of the experiment (Table 2).

**TABLE 1:** Brain perfusion, oxygenation, microdialysis-derived metabolism and mitochondrial respiration.

Parameter	Brain hemisphere	Group	Baseline	2 h ASDH + HS	24 h treatment	48 h treatment
Numbers of animals alive		C	7	7	5	3
		T	7	7	6	6
	ASDH	C	35.4 (35.2; 35.6)	35.9 (35.4; 36.5)	38.5 (38.1; 39.3)#	38.4 (38.1; 38.6)#
Brain		T	35.1 (34.4; 35.7)	35.4 (33.9; 36.3)	37.9 (37.3; 38.5)#	37.9 (37.4; 38.3)#
Temperature [°C]	Contra-lateral	C	35.4 (35.1; 35.8)	35.8 (35.7; 36.4)	38.5 (38.3; 39.2)#	38.2 (37.9; 38.7)#
		T	35.1 (34.5; 36)	35.3 (34.1; 36.3)	38.0 (37.3; 38.4)#	37.9 (37.3; 38.3)#
	ASDH	C	10 (5; 13)	15 (2; 20)	14 (5; 58)	24 (19; 88)
Intracranial		T	12 (6; 16)	10 (4; 12)	24 (15; 31)#	24 (23; 32)
Pressure [mmHg]	Contra-lateral	C	7 (4; 9)	10 (5; 15)	15 (9; 32)#	4 (1; 11)
		T	11 (7; 15)	12 (7; 18)	24 (18; 27)#	23 (22; 35)#§
	ASDH	C	98 (83; 133)	59 (56; 65)#	99 (44; 102)	73 (59; 88)
Cerebral perfusion		T	112 (95; 117)	62 (52; 69)#	86 (71; 99)	84 (73; 103)
Pressure [mmHg]	Contra-lateral	C	96 (91; 142)	56 (50; 68)#	94 (76; 98)	103 (72; 123)
		T	109 (96; 119)	59 (51; 63)#	91 (72; 98)	82 (70; 104)#
	ASDH	C	2.9 (2.1; 3.8)	4.1 (3.5; 5.0)	4.7 (2.7; 4.8)	2.2 (1.6; 3.4)
Microdialysis		T	2.9 (1.5; 3.4)	3.2 (3.1; 4.4)	3.0 (2.9; 4.3)	2.0 (1.6; 2.9)
Lactate [mmol/L]	Contra-lateral	C	2.3 (1.4; 3.0)	3.1 (2.6; 3.4)	2.3 (1.4; 3.0)	1.3 (0.8; 1.7)
		T	1.9 (1.6; 3.5)	2.2 (1.5; 2.3)	2.9 (2.4; 3.7)	2.3 (1.8; 4.1)
	ASDH	C	22 (15; 30)	38 (20; 42)	52 (33; 81)	44 (12; 131)
Microdialysis		T	29 (26; 38)	30 (25; 359	61 (22; 65)	31 (21; 52)
Pyruvate [μmol/L]	Contra-lateral	C	37 (20; 42)	34 (17; 56)	30 (9; 46)	27 (8; 84)
		T	24 (15; 59)	36 (19; 40)	20 (16; 25)	39 (20; 48)
	ASDH	C	0.45 (0.25; 0.66)	0.52 (0.11; 0.69)	0.03 (0.02; 0.18)	0.33 (0.01; 0.85)
Microdialysis		T	0.33 (0.22; 0.62)	0.24 (0.18; 0.55)	0.04 (0.01; 0.14)#	0.03 (0.02; 0.06)#§
Glucose [mmol/L]	Contra-lateral	C	1.24 (0.38; 1.90)	1.35 (0.28; 1.78)	0.03 (0.02; 0.52)	0.31 (0.02; 0.99)
		T	0.43 (0.19; 0.62)	0.49 (0.15; 0.82)	0.08 (0.02; 0.14)#	0.02 (0.01; 0.05)#
	ASDH	C	56 (28; 64)	36 (20; 42)	59 (18; 82)	20 (12; 44)
Microdialysis		T	39 (34; 72)	35 (33; 58)	16 (12; 24)#	11 (7; 12)#
Glutamate [μmol/L]	Contra-lateral	C	40 (21; 63)	26 (16; 29)	33 (10; 65)	22 (9; 51)
		T	49 (43; 49)	31 (25,42)	17 (16; 26)#	23 (18; 27)#

Data are median (interquartile range), # denotes p < 0.05 vs. baseline within a group, § denotes p < 0.05 vs. control. ASDH: blood-injected hemisphere; Contralateral: sham-instrumented hemisphere (grey, C = control; purple, T = treatment/hyperoxemia). At 24 h of “TBI-targeted ICU care” two control animals had already died (see Figure 1). However, since these animals had to be euthanized less than 2 h before the scheduled time point at 24 h of ICU care, the respective immediate pre-mortal values recorded at that time point were used as readings for “24 hours treatment.” Due to one drop-out because of uncontrollable bleeding during the surgical instrumentation data refer to 14 animals (n = 7 per group).

**TABLE 2:** Assessment of the veterinary Modified Glasgow Coma Scale.

Parameter	Group	Baseline	2 h ASDH + HS	24 h treatment	48 h treatment
Numbers of animals alive	C	7	7	5	3
	T	7	7	6	6
Veterinary Modified Glasgow Coma Scale	C	16 (16; 18)	n.d.	4 (3; 16) #	3 (3; 15) #
	T	14 (11; 17)	n.d.	14 (12; 17)	13 (3; 17)

Data are median (interquartile range), # denotes p < 0.05 vs. baseline within a group. All experiments that terminated pre-schedule were terminated due to sudden ICP increases and a consecutive and refractory fall of CPP < 60 mmHg. Therefore, to extrapolate the “missing values” at the respective time points, in these animals the minimum value possible for the Veterinary Modified Glasgow Coma Scale (= 3) was used (grey, C = control; purple, T = treatment/hyperoxemia). At 24 h of “TBI-targeted ICU care” two control animals had already died (see Figure 1). However, since these animals had to be euthanized less than 2 h before the scheduled time point at 24 h of ICU care, the respective immediate pre-mortal values recorded at that time point were used as readings for “24 hours treatment”.

### Biomarkers of inflammation, oxidative stress and brain injury

Table 3 summarizes the plasma biomarkers of inflammation, oxidative stress and brain injury. While IL-6 concentrations decreased over time without intergroup difference, IL-10 levels showed the opposite pattern, however, without intergroup difference. Hyperoxemia did not aggravate oxidative stress as evaluated by the superoxide anion and isoprostane concentrations (Table 3), which was confirmed by the unaffected mitochondrial respiratory activity (both oxidative phosphorylation and maximum electron transport capacity of the respiratory chain in the uncoupled state) in *post mortem* brain specimen (Supplementary Table 2). Plasma concentrations of all brain injury biomarkers (GFAP, MAP-2, NSE, and protein S100β) progressively decreased in the hyperoxemia group, whereas MAP-2 only showed this pattern in the control animals (Table 3).

**TABLE 3:** Biomarkers of inflammation, oxidative stress, and brain injury.

Parameter	Group	Baseline	2 h ASDH + HS	24 h treatment	48 h treatment
Numbers of animals alive	C	7	7	5	3
	T	7	7	6	**6**
Interleukin-6	C	2108 (1804; 3847)	1324 (1078; 1760)	356 (132; 666)	93 (46; 448) *p* = 0.07 within group
[ng/L]	T	2163 (638; 2613)	677 (336; 1091)	232 (196; 434)	91 (83; 122) #
Interleukin-10	C	16 (11; 22)	50 (42; 57) #	28 (22; 34)	32 (32; 44)
[ng/L]	T	15 (12; 18)	44 (42; 75)	25 (18; 28)	27 (16; 55) *p* = 0.06 within group
Superoxide anion	C	8.6 (6.6; 20.7)	7.0 (6.1; 17.4)	6.7 (6.4; 8.5)	10.6 (7.4; 18.1)
[μmol/L]	T	6.5 (5.8; 8.5)	6.3 (5.3; 9.9)	6.6 (6.3; 7.7)	7.4 (6.3; 7.8)
8-isoprostane	C	91 (67; 101)	98 (78; 123)	123 (69; 267)	51 (46; 52) *p* = 0.05 within group
[ng/L]	T	71 (51; 76)	53 (44; 72)	60 (46; 141)	43 (31; 52)
GFAP	C	33 (23; 41)	31 (20; 38)	16 (8; 24)	22 (18; 30)
[ng/L]	T	24 (15; 27)	19 (14; 22)	9 (9; 10)#	11 (10; 21)#
MAP-2	C	3.1 (2.3; 4.6)	3.1 (1.9; 4.5)	1.2 (0.7; 1.6)#	1.1 (0.8; 2.3)
[μg/L]	T	2.4 (1.9; 2.9)	1.6 (1.1; 2.2)	0.7 (0.3; 0.7)#	0.9 (0.7; 1.3)#
NSE	C	33 (23; 41)	31 (20; 38)	16 (8; 24)	22 (18; 30)
[μg/L]	T	24 (15; 28)	19 (14; 22)	9 (9; 10)#	11 (10; 21)#
Protein S100β	C	1.2 (0.8; 2.3)	0.9 (0.6; 1.7)	0.8 (0.4; 1.1)	0.7 (0.6; 1.2)
[μg/L]	T	0.3 (0.3; 0.5) §	0.3 (0.2; 0.9)	0.3 (0.2; 0.4) *p* = 0.05 vs. *Control*	0.3 (0.2; 0.3)

All data are median (interquartile range), # denotes p < 0.05 vs. baseline within a group, §  denotes p < 0.05 vs. control. GFAP, glial fibrillary acidic protein; MAP-2, microtubule-associated protein 2; NSE, neuron- specific enolase; Results of the assessment of the Veterinary Modified Glasgow Coma Scale. Data are median (interquartile range), # denotes p < 0.05 vs. baseline within a group. All experiments that terminated pre-schedule were terminated due to sudden ICP increases and a consecutive and refractory fall of CPP < 60 mmHg. Therefore, to extrapolate the “missing values” at the respective time points, in these animals the minimum value possible for the Veterinary Modified Glasgow Coma Scale (= 3) was used (grey, C = control; purple, T = treatment/hyperoxemia). At 24 h of “TBI-targeted ICU care” two control animals had already died (see Figure 1). However, since these animals had to be euthanized less than 2 h before the scheduled time point at 24 h of ICU care, the respective immediate pre-mortal values recorded at that time point were used as readings for “24 hours treatment”.

## Discussion

The main results of this study were that in a long-term, resuscitated, animal model of combined ASDH and HS over 56 h post-injury, targeted hyperoxemia (i) ameliorated brain tissue oxygenation, which persisted beyond the intervention period; (ii) attenuated neurologic dysfunction; and (iii) was safe with respect to parameters of oxidative stress. This porcine model mimics a frequent scenario of severe combined injury, namely hemorrhagic shock and ASDH, the latter being a major contributor to TBI (14). The model makes use of “FBM” swine characterized by atherosclerosis and coronary artery disease, and, thus, mirrors the elderly and co-morbid patient (15).

While it seems unequivocal that excessive hyperoxemia with P_a_O_2_ > 300 mmHg should be avoided (3, 4), it remains open whether there is a “*sweet spot*” for P_a_O_2_ after TBI and/or HS (3, 4). This discussion is best characterized that for possible neuroprotective properties of hyperox(em)ia, there is “… *probably a narrow effective dose, and benefit may be limited to at-risk tissue*…” (25), and the “ … *need to identify optimal approaches to improve O_2_ delivery without exacerbating*… *oxidative stress or injury*…” (26). Our findings of possible beneficial effects of a target window of P_a_O_2_ = 200–250 mmHg during the first 24 h of resuscitation agree with retrospective data after severe multiple trauma showing that P_a_O_2_ > 150 and > 200 mmHg, respectively, within the first 24 h of ICU stay coincided with improved survival (7, 8). The interval chosen in our study was also associated with the lowest mortality and the best neurological outcome after severe TBI during the first 24 h of ICU (9). It is noteworthy that albeit not significantly different, the two survival curves cleaved at approx. 24 h of resuscitation (*p* = 0.11). Again, this agrees with retrospective data in TBI patients concluding that moderate hyperoxemia with “…*a P_a_O_2_ above 12 kPa and higher may improve oxidative cerebral energy metabolism and pressure autoregulation, particularly in cases of limited energy substrate supply in the early phase of TBI*…” (27).

It could be argued that hyperox(em)ia-induced systemic vasoconstriction should have caused reduced norepinephrine infusion rates (3, 4). Moreover, due to its preferential vasoconstrictor effect in the cerebral circulation, target hyperox(em)ia might have been expected to lower ICP values (3, 4). It should be noted, however, that in the HYPER2S multicenter trial in patients with norepinephrine-dependent septic shock, even 24 h of F_I_O_2_ = 1.0 resulting in mean P_a_O_2_ levels of 227–272 mmHg during the treatment period had not influenced the norepinephrine infusion rates (28). In mechanically ventilated patients with severe TBI and requiring F_I_O_2_ levels < 0.4 to maintain normoxemia, normobaric hyperoxia (F_I_O_2_ = 1.0) resulting in P_bt_O_2_ levels of 86 ± 12 mm Hg, i.e., even slightly higher than in our study, had not affected ICP (29). Hence, our findings well agree with existing clinical data.

P_bt_O_2_ monitoring has been referred to as a promising tool after TBI, in particular when combined with ICP measurements and/or microdialysis data (30). In contrast to our study, Hawryluk et al. reported that in swine undergoing controlled cortical impact, hyperoxia increased P_bt_O_2_ values distal to, but if so, had only little effect near the site of injury. It should be noted, however, that these authors’ study lasted for 15.2 h only, animals received no hemodynamic support to maintain perfusion pressure, and F_I_O_2_ = 100% was used, with P_a_O_2_ as high as ≈ 560 mmHg (31). Albeit P_bt_O_2_ correlates with P_a_O_2_ (32, 33), equivocal results are available whether a hyperoxemia-induced P_bt_O_2_ increase improves cerebral energy metabolism (34–37). Strikingly, the effect of targeted hyperoxemia on P_bt_O_2_ values was sustained until the end of our experiment, i.e., beyond the intervention period. It could be argued that increased microcirculatory O_2_ content under conditions of comparable macrocirculatory O_2_ availability might reflect impaired cellular O_2_ utilization rather than improved energy metabolism. However, albeit there was no significant intergroup difference, microdialysis data favored the hyperoxemia-treated animals: glutamate concentrations, a marker of excitotoxic secondary brain injury in patients (38) and swine (39, 40), progressively declined in the hyperoxemia-group, while they remained unchanged in the control group. At first glance, this finding is in contrast to retrospective patient data demonstrating a *direct* relation between hyperoxia and microdialysis-derived glutamate concentrations, i.e., suggesting hyperoxia-induced aggravation of excitotoxicity (41). However, in that study, increased glutamate concentrations under conditions of “*compromised”* P_bt_O_2_ values (< 20 mmHg, i.e., comparable to those in our control animals) required an F_I_O_2_ > 60%. Since in that study the “…*majority of patients had no pulmonary dysfunction*…,” P_a_O_2_ levels had most likely been markedly higher than in our swine with maximum F_I_O_2_ = 40%.

Targeted hyperoxemia affected neither blood O_2⋅_^–^ (a marker of ROS formation) nor isoprostane (a marker of lipid peroxidation) concentrations. Immediate *post mortem* brain tissue mitochondrial respiration was not affected either. Both in experimental animals and in patients, equivocal data is available on the impact of hyperox(em)ia on oxidative stress after TBI: aggravated (42), unchanged (43, 44) or attenuated (45) markers of radical damage were reported. In mice with TBI and HS, Blasiole et al. found that despite enhanced oxidative stress and neuro-inflammation, hyperoxic resuscitation improved hippocampal neuronal survival (46). The authors concluded that benefits of enhanced O_2_ supply may outweigh detrimental effects of enhanced radical production. It is noteworthy that most of the above-mentioned studies investigated short-term (up to 4 h) pure O_2_ ventilation with P_a_O_2_≈300–450 mmHg rather than moderate hyperoxemia within a well-defined target window. According to the clinical data available (9) we had avoided extreme hyperoxemia: even hyperoxemia comparable to our study for up to 14 days to did not affect serum markers of oxidative stress, inflammation or neurological injury (44).

### Limitations

The major limitation of this study is that we used MGCS as the main outcome parameter, but that a power analysis had not been possible prior to the study due to the lacking available data on the time course of this parameter in a model of combined ASDH plus hemorrhage. Therefore, we had been incapable to calculate a number of animals to include to observe a difference in MGCS, and, consequently, the number of animals studies was limited, rendering the study merely exploratory. Moreover, the experimental setting clearly is established for either ASDH or HS alone (10, 17), but no comparable data are available in the current literature for the combined trauma. Finally, the approach is limited due to its duration of 56 h of injury and resuscitation for reasons of ethical acceptability and feasibility.

## Conclusion

In summary, in a resuscitated model of combined ASDH and HS over 56 h post-injury in swine with pre-existing atherosclerosis target hyperoxemia was associated with higher P_bt_O_2_ levels, which was sustained beyond the intervention period. Target hyperoxemia exerted neither significantly beneficial nor deleterious effects. A benefit for neurological outcome may have been missed due to the tendency toward lower survival in the normoxemia-treated animals.

## Data availability statement

The original contributions presented in this study are included in the article/Supplementary material, further inquiries can be directed to the corresponding author.

## Ethics statement

The animal study was reviewed and approved by Ethical approval by the Animal Care Committee of the Universität Ulm and the Federal Authorities for Animal Research (Regierungspräsidium Tübingen, #1316) experiments were conducted in adherence to the European Union Directive 2010/63/EU on the protection of animals used for scientific purposes.

## Author contributions

PR and TK: conceptualization. PR, TK, and AH: methodology. MG, FZ, OM, TM, E-MW, EC, TD, DM, and PR: formal analysis. TD, DM, HG, AH, MG, RM, SM, TT, SZ, EC, PR, and TK: investigation. PR: resources and supervision. TD and PR: writing – original draft. TD, DM, FM, AH, MG, RM, SM, HG, FZ, OM, TM, AS, E-MW, TT, SZ, EC, PA, PR, and TK: writing – review and editing. TD, DM, TT, and SZ: visualization. All authors have read and approved the final version of the article.
