# Supplementary material for: The effect of targeted hyperoxemia in a randomized controlled trial employing a long-term resuscitated, model of combined acute subdural hematoma and hemorrhagic shock in swine with coronary artery disease: An exploratory, hypothesis-generating study
Source: Front Med (Lausanne). 2022 Aug 22;9:971882. doi: 10.3389/fmed.2022.971882 (PMC9442904; doi:10.3389/fmed.2022.971882)
Supplement: Supplementary file 1 [file Data_Sheet_1.docx]

Supplementary Material

# Supplementary Tables

## Supplemental Table 1

| *Parameter* | *Group* | **baseline** | **2 hours**  **ASDH + HS** | **24 hours treatment** | **48 hours treatment** |
| --- | --- | --- | --- | --- | --- |
| Body temperature | C | 35.5 (35.2;35.9) | 35.8 (35.6;36.6) | 38.8 (37.9;39.2) **#** | 38.7 (38.3;38.8) **#** |
| [°C] | T | 35.3 (34.6;35.9) | 35.8 (34.6;36.4) | 38 (37.4;38.7) **#** | 38.1 (37.5;38.2) **#** |
| Heart rate | C | 50 (43;61) | 139 (117;142) | 83 (79;153) | 111 (82;147) |
| [1/min] | T | 59 (44;73) | 139 (108;157) | 78 (67;110) | 84 (75;110) |
| Mean arterial | C | 103 (92;146) | 66 (63;74) **#** | 109 (96;113) | 107 (82;124) |
| pressure [mmHg] | T | 124 (103;127) | 71 (63;76) **#** | 109 (96;126) | 114 (106;131) |
| Venous pressure | C | 6 (3;8) | 1 (0;3) **#** | 9 (6;11) | 5 (3;7) |
| [mmHg] | T | 6 (5;7) | 1 (1;4) **#** | 11 (6;15) | 11 (7;16) |
| Cardiac output | C | 4.9 (4.8;5.2) | 3.3 (2.4;3.5) **#** | 5.5 (4.8;11.8) | 5.9 (5.5;6.0) |
| [L/min] | T | 5.0 (4.5;5.8) | 2.8 (2.5;3.4) **#** | 5.3 (4.9;7.2) | 5.4 (5.1;6.7) |
| Stroke volume | C | 99 (85;111) | 25 (18;27) **#** | 64 (56;72) **#** | 53 (50;82) **#** |
| [mL] | T | 88 (76;95) | 23 (18;27) **#** | 73 (64;90) **#** | 65 (59;78) **#** |
| Pulse pressure | C | 6 (5;10) | 19 (13;22) **#** | 17 (14;17) **#** | 15 (12;25) **#** |
| variation [%] | T | 8 (4;12) | 22 (18;28) **#** | 8 (7;24) | 11 (10;16) |
| Stroke volume | C | 16 (10;16) | 18 (17;20) **#** | 13 (7;22) | 24 (10;28) **#** |
| variation [%] | T | 8 (8;11) | 21 (15;34) **#** | 10 (9;21) | 16 (14;23) **#** |
| Total hemoglobin | C | 10.4 (8.2;11.7) | 10.5 (9.8;11.6) | 10 (7.6;10.9) | 9.8 (5.9;11.1) |
| content [g/dL] | T | 9.2 (8.5;9.5) | 8.8 (8.3;10) **§** | 8.6 (7.9;11.3) | 7.9 (6.1;9.2) **#** |
| O_2_ uptake | C | 153 (136;235) | 153 (139;226) | 252 (210;257) **#** | 218 (208;356) **#** |
| [mL/min] | T | 171 (147;208) | 163 (138;218) | 301 (247;326) **#** | 289 (244;360) **#** |
| CO_2_ production | C | 111 (103;199) | 119 (106;182) | 213 (157;242) **#** | 174 (147;216) **#** |
| [mL/min] | T | 156 (114;204) | 140 (123;191) | 184 (156;223) **#** | 165(149;194) |
| Arterial PO_2_ | C | 100 (88;105) | 97 (93;103) | 106 (91;138) | 101 (85;105) |
| [mmHg] | T | 105 (82;117) | 102 (89;115) | 216 (204;233) **#§** | 106 (96;110) |
| Arterial PCO_2_ | C | 39 (32;42) | 37 (32;44) | 40 (35;41) | 39 (38;43) |
| [mmHg] | T | 39 (35;39) | 36 (33;44) | 40 (37;44) | 38 (37;39) |
| Arterial pH | C | 7.48 (7.45;7.51) | 7.48 (7.40;7.52) | 7.51 (7.45;7.54) | 7.54 (7.51;7.56) **#** |
|  | T | 7.50 (7.45;7.51) | 7.48 (7.42;7.54) | 7.51 (7.43;7.53) | 7.54 (7.52;7.59) **#** |
| Arterial base | C | 4.6 (2.7;5.4) | 2.4 (1.8;3.0) **#** | 8.3 (-1.4;9.6) | 10.1 (7.2;12.4) **#** |
| excess [mmol/L] | T | 4.8 (3.5;5.3) | 4.3 (2.2;5.3) | 7.1 (1.9;89.3) | 9.5 (7.5;11.4) **#** |
| Arterial lactate | C | 2.7 (2.4;3.1) | 3.9 (2.9;4.0) **#** | 2.4 (0.9;5.3) | 1.2 (0.7;1.6) **#** |
| [mmol/L] | T | 2.8 (2.4;3.4) | 4.7 (4.0;5.2) **#** | 2.7 (1.1;7.2) | 1.2 (1.0;1.5) **#** |
| Arterial glucose | C | 91 (86;96) | 92 (78;113) | 88 (80;111) | 90 (89;111) |
| [mg/dL] | T | 81 (62;92) | 70 (64;79) **§** | 80 (69;101) | 83 (74;100) |
| Arterial Na^+^ | C | 143 (142;145) | 142 (140;144) | 144 (142;147) | 146 (145;147) |
| [mmol/L] | T | 145 (144;146) | 144 (142;145) | 142 (142;146) | 141 (139;146) |
| Arterial K^+^ | C | 3.1 (3.0;3.2) | 3.1 (3.1;3.3) | 2.9 (2.8;3.2) | 3.1 (2.9;3.1) |
| [mmol/L] | T | 3.0 (2.9;3.1) | 3.2 (3.1;3.4) | 3.1 (2.9;3.4) | 3.1 (2.9;3.3) |
| Arterial urea | C | 4.6 (3.9;5.2) | 5.4 (4.7;5.6) | 2.8 (2.5;3.4) **#** | 2.1 (2.0:2.2) |
| [mmol/L] | T | 5.7 (4.7;5.9) | 5.7 (5.5;6.3) | 3.3 (2.8;3.7) **#** | 2.9 (2.7;3.0) **#** |
| Calculated | C | 303 (302;304) | 301 (298;302) | 302 (296;309) | 305 (300;308) |
| osmolarity [mmol/L] | T | 308 (303;308) | 3030 (300;305) | 300 (297;306) | 294 (288;297) |
|  |  |  |  |  |  |
| Amount of blood | C | 25 (19; 27) | | | |
| withdrawn [mL/kg] | T | 25 (18; 28) | | | |
| Noradrenaline | C | 1.13 (0.69; 2.42) # | | | |
| [µg kg^-1^ min^-1^] | T | 1.77 (0.52; 2.24) # | | | |
| Diuresis | C |  |  | 5.9 (4.3; 8.2) | 10.1 (8.1; 10.8) |
| [mL kg^-1^ h^-1^] | T |  |  | 9.5 (3.6; 9.5) | 10.2 (8.66; 15.3) |

**Supplemental Table 1**: **Systemic hemodynamics, gas exchange, acid-base-status, and metabolism.** All data are median (interquartile range), **#** denotes p < 0.05 *vs.* baseline within a group, **§** denotes p < 0.05 *vs.* control. Osmolarity was calculated according to the formula *(Na^+^ + K^+^) · 2 + Urea + Glucose/18* (yellow, C = control; purple, T = treatment/hyperoxemia)*.*

## Supplemental Figure 2

| Brain Hemisphere | *Group* | **OxPhos** | **ETC** |
| --- | --- | --- | --- |
| ASDH | C | 7.6 (6.7;17.0) | 11.1 (8.9;21.4) |
|  | T | 6.7 (6.5;20.7) | 10.3 (10.2;33.6) |
| Contralateral | C | 8.6 (7.4;11.1) | 11.8 (10.4;14.9) |
|  | T | 7.7 (6.9;12.4) | 11.2 (10.5;17.6) |

**Supplemental Table 2: Mitochondrial respiration (J*O_2_* [pmol sec ^-1^ g_protein_^-1^]) in immediate *post mortem* brain specimen.** Data are median (interquartile range). ASDH blood-injected hemisphere; Contralateral sham-instrumented hemisphere; OxPhos oxidative phosphorylation, ETC electron transport capacity of the respiratory chain in the uncoupled state (yellow, C = control; purple, T = treatment/hyperoxemia). Mitochondrial respiration was measured in homogenized tissue samples from the ipsi- and contralateral cortex by means of high-resolution respirometry using the Oroboros Oxygraph-2K (Oro-boros Instruments, Innsbruck, Austria). This device allows to simultaneously record the O_2_-concentration in two parallel chambers calibrated for 2mL of respiration medium comprising sucrose, K-lactobionate, ethylene glycol tetra acetic acid, bovine serum albumin free from essentially fatty acids, MgCl_2_, taurine, KH2PO4, HEPES, adjusted to pH 7.1 with KOH and equilibrated with 21% O_2_ in N_2_ at 37 ̊C. Mitochondrial respiration was quantified in terms of oxygen flux (JO_2_) based on the rate of change of the O_2_-concentration in the chambers normalized for wet tissue volume. The tissue homogenates were generated from 15–20mg of brain tissue suspended in 2mL of ice-cold respiration medium using a potter S homogenizer (B. Braun, Melsungen, Germany). Aliquots of the homogenates were added to each oxygraph chamber in order to obtain a standardized final amount of 2mg tissue per chamber. Every sample was measured in duplicates and normalized to the amount of tissue per chamber. Maximum mitochondrial respiration is achieved by stepwise injection of substrates into the two chambers of the oxygraph, according to well established and previously published protocols.(1, 2) The titration sequence used for the current experiment was as follows: 5mM malate, 10mM glutamate, 5mM ADP, 10μM cytochrome c, 10mM pyruvate, 10mM succinate 5μM oligomycin, 0.5μM carbonyl cyanide p-(trifluoromethoxy)-phenylhydrazone (FCCP), 0.5μM rotenone and 5μM antimycin A. The maximum oxidative capacity of the mitochondrial respiratory chain in the uncoupled condition was determined after the final steps consisting in the addition of the complex II substrate succinate, followed by the subsequent injections of the ATP synthase inhibitor oligomycin and of the uncoupler FCCP.

1 Eva Buck et al. High-resolution respirometry of fine-needle muscle biopsies in pre-manifest Huntington's disease expansion mutation carriers shows normal mitochondrial respiratory function. PLoS One. 2017 Apr 13;12(4): e0175248. doi: 10.1371/journal.pone.0175248. eCollection 2017.

2 Ivonne Heinze et al. Species comparison of liver proteomes reveals links to naked mole-rat longevity and human aging. BMC Biology (2018) 16:82 https://doi.org/10.1186/s12915-018-0547-y
